# Using Functional Near-Infrared Spectroscopy to Study the Effect of Repetitive Transcranial Magnetic Stimulation in Concussion: A Two-Patient Case Study

**DOI:** 10.3389/fneur.2019.00476

**Published:** 2019-05-08

**Authors:** Joan M. Stilling, Chris C. Duszynski, Ibukunoluwa Oni, Eric Paxman, Jeff F. Dunn, Chantel T. Debert

**Affiliations:** ^1^Hotchkiss Brain Institute, Calgary, AB, Canada; ^2^Department of Clinical Neurosciences, Cumming School of Medicine, University of Calgary, Calgary, AB, Canada; ^3^Department of Radiology, Cumming School of Medicine, University of Calgary, Calgary, AB, Canada

**Keywords:** concussion, post-concussion symptom, transcranial magnetic stimulation (repetitive), functional Near Infrared Spectroscopy (fNIRS), rehabilitation

## Abstract

**Background:** Approximately 25% of concussion patients experience persistent post-concussion symptoms (PPCS). Repetitive transcranial magnetic stimulation (rTMS) has been explored as a treatment, and functional near-infrared spectroscopy (fNIRS) may be a cost-effective method for assessing response.

**Objectives:** Evaluate rTMS for the treatment of PPCS and introduce fNIRS as a method of assessing treatment response.

**Methods:** *Design*: Two-patient case study. *Setting:* Calgary Brain Injury Program. *Participants:* 47 and 49 years. male, with PPCS for 1–2 years (headache, cognitive difficulties, nausea, visual difficulties, irritability, anxiety, poor mood, sleep, and fatigue). *Intervention:* 10 sessions of rTMS therapy to the left dorsolateral prefrontal cortex (DLPFC), at 10 Hz (600 pulses) and 70% of resting motor threshold amplitude. Participants completed an 8-week headache diary and a battery of clinical questionnaires prior to each fNIRS session. *fNIRS:* Hemodynamic changes were recorded over the frontoparietal cortex during rest, finger tapping, and a graded working memory test. fNIRS was completed pre-rTMS, following rTMS (day 14), and at 1-month post-rTMS (day 45). For comparison, two healthy, sex-matched controls were scanned with fNIRS once daily for five consecutive days.

**Results:** Clinical scores improved (headache severity, MoCA, HIT-6, PHQ-9, GAD-7, QOLIBRI, RPSQ, BCPSI) or remained stable (PCL-5, headache frequency) post-rTMS, for both participants. Participant 1 reported *moderate* symptom burden, and a fNIRS task-evoked hemodynamic response showing increased oxyhemoglobin was observed following a working memory task, as expected. Participant 2 exhibited a *high* symptom burden pre-treatment, with *abnormal* fNIRS hemodynamic response where oxyhemoglobin declined, in response to task. One month following rTMS treatment, participant 2 had a normal fNIRS hemodynamic response to task, corresponding to significant improvements in clinical outcomes.

**Conclusion:** This case study suggests fNIRS may be sensitive to physiological changes that accompany rTMS treatment. Further studies exploring fNIRS as a cost-effective technology for monitoring rTMS response in patients with PPCS are suggested.

## Background

Annually, up to 280,000 people in Canada (1) and 42 million worldwide (2, 3) experience a mild traumatic brain injury (mTBI). In patients with mTBI, symptoms experienced following injury usually resolve within 3 months. However, up to 25% of patients will experience persistent post-concussion symptoms (PPCS), which can continue up to 1 year following injury (4). Common symptoms include headaches, dizziness, fatigue, irritability, depression, anxiety, emotional lability, concentration or memory difficulties, insomnia, and reduced alcohol tolerance (ICD-10 post-concussion syndrome diagnostic criteria) (5–8).

Repetitive transcranial magnetic stimulation (rTMS) is a non-invasive neurostimulation treatment whereby a rapidly alternating magnetic field applied to the scalp induces an electrical stimulus in a targeted region of the brain (9). This may lead to neuronal depolarization and either excitation or inhibition, depending on the neurons stimulated. Clinically, TMS has been approved by the FDA for treatment-resistant depression (rTMS) (10, 11) and migraine with aura (single pulse TMS) (12) Recent preliminary studies have explored rTMS as a treatment option for PPCS (13–15), although the physiological changes associated with rTMS intervention remain relatively unknown.

Functional near infrared spectroscopy (fNIRS) is a neuroimaging technology that non-invasively measures changes in cerebral tissue oxygenation coupled to neuronal activity. Continuous traces of cerebral tissue oxygenation are recorded and changes in the optical absorption properties of the brain tissue are measured (16). These changes can then be used to map local changes in brain activity, similar to how the blood-oxygenation level-dependent (BOLD) signal is used to measure brain activity in functional MRI studies (17). In comparison to functional MRI, fNIRS is advantageous for clinical application because it is small, portable, and useful in a variety of environments where neuroimaging is not feasible. We propose functional near-infrared spectroscopy (fNIRS) as a cost-effective method for studying rTMS treatment response. In this case study, we utilized fNIRS to explore the relationship between physiological changes in brain function and clinical markers of recovery associated with rTMS treatment in two patients with PPCS.

## Objectives

To evaluate the effectiveness of rTMS for the treatment of PPCS and to assess whether fNIRS could be a biomarker of rTMS treatment response.

## Patient Histories

### Participant #1

A 47-year-old male accountant was seen in the Calgary Brain Injury Program (CBIP) at the Division of Physical Medicine and Rehabilitation Department, Foothills Medical Centre, Calgary, AB, Canada. He was originally referred with a history of persistent symptoms (headache, dizziness, vision, neck pain, nausea, poor sleep, and mood) following mild traumatic brain injury while playing soccer. He had a past medical history of migraine with aura, basal cell carcinoma (removed), and gastroesophageal reflux disorder (GERD).

The patient was hit in the head by a high-speed ball while playing in a soccer game. He did not lose consciousness nor report post traumatic amnesia. He finished playing the game and was later diagnosed with a sport-related concussion (SRC) by a sports medicine physician based on the Consensus statement on Concussion in sport−5th International Conference (18). Initial symptoms included feeling “off and foggy” for the first 3 days, with subsequent development of persisting headaches/head pressure sensations, vision difficulties, fatigue, slowed processing speed and dizziness. Approximately 2–3 weeks after the head injury, he had a CT scan of his head, which was normal. In regard to treatment, he had trialed craniosacral therapy, physiotherapy, vestibular and vision therapy with only mild improvement in his symptoms. As a result, he was on short term disability.

Physical exam at the initial assessment demonstrated evidence of saccades when looking to the left and left eye nystagmus when looking to the right. He had evidence of convergence insufficiency on exam. The remainder of his neurologic exam was normal. Investigations, including neuro-ophthalmology evaluation, CT head, and neuroendocrine testing (CBC, electrolytes, glucose, TSH, free T4, a.m. cortisol) were all within normal limits.

The patient was seen seven times at the CBIP over the course of 16 months with ongoing treatment for headache, neck pain/hyperalgesia, vision, mood and return to work counseling. Despite further treatment with oral pharmacologic medications (trazadone, amitriptyline, desvenlafaxine), topicals, greater occipital nerve blocks, cranial botox injections (PREEMPT protocol) (19), prism glasses, and exercise, he continued to experience PPCS.

### Participant #2

A 49-year-old male elementary school teacher was originally referred to the CBIP with a history of PPCS (vision changes, headaches, confusion, slowed thinking, difficulty with multitasking, poor balance, postural dizziness, nausea, emotional lability, fatigue) following a motor vehicle accident. He had a past medical history of a mild TBI (with loss of consciousness) at 19 years old, as well as remote history of lower extremity orthopedic injuries.

The patient was involved in a motor vehicle accident, which occurred at ~50 km/h. The air-bags deployed and his car started on fire. He was unable to remove himself from the vehicle. He did not report amnesia or loss of consciousness, but did experience a sensation of nausea, vision changes, headache, dizziness, and fatigue following the event. He was diagnosed with a concussion by his family doctor in accordance with the World Health Organization Criteria (20), based on alteration in mental state and neurological deficits following the event. He tried to go back to work 3 days after the injury, however reported word-finding difficulties, problems concentrating, poor memory, and confusion. Treatment prior to assessment in the CBIP included acetaminophen, ibuprofen, physiotherapy, massage, and hyperbaric oxygen chamber therapy without significant benefit. The patient was on short-term disability from work as a Grade 5 teacher. He was previously active in Iron Man Triathlons but was unable to train since his accident.

Initial physical exam demonstrated convergence insufficiency. He became symptomatic with eye movement. The rest of his neurologic exam was normal. Investigations, including an MRI of the brain and neuroendocrine testing (CBC, electrolytes, glucose, TSH, free T4, IGF-1, a.m. cortisol, and urine electrolytes) were within normal limits.

He was seen five times at CBIP over the course of 8 months with ongoing treatment for vision, headaches, dizziness, mood, return to exercise, and general rehabilitation (occupational and physical therapy, social work, psychology, and productivity consultant). He was treated with oral medications (sertraline, rizatriptan), prism glasses, vestibular physiotherapy, vision therapy, and personal exercise training with ongoing post-concussion symptoms.

## Methods

### Design

Two-patient case study.

### Setting

Calgary Brain Injury Program, Calgary, Alberta, Canada.

### Included Participants

The two included participants, described above, consented to participate in a case study using rTMS and fNIRS for possible treatment of their persistent post traumatic brain injury symptoms. Two male control subjects (19 and 28 years of age, respectively) with no history of concussion or mild TBI underwent five identical fNIRS scans across five consecutive measurement days, in an effort to demonstrate reproducibility of baseline fNIRS measurement. Participant 1 was maintained on oral pharmacologic management with amitriptyline throughout the trial, while participant 2 did not take any oral medications.

### Intervention

Ten sessions of rTMS therapy using an air-cooled 70-mm coil (Airfilm; Magstim, Whitland, UK) to the left dorsolateral prefrontal cortex (DLPFC), over 2 weeks, at 10 Hz (600 pulses) and 70% of resting motor threshold amplitude. Participants clinical MRI scans were loaded onto the neuronavigation software platform (Brainsight2, Rogue, Montreal).

### Assessments

Clinical questionnaires included the headache impact test-6 (HIT-6), Montreal cognitive assessment (MoCA), patient health questionnaire-9 (PHQ-9), generalized anxiety disorder scale-7 (GAD-7), post-traumatic stress disorder checklist for DSM-5 (PCL-5), quality of life after brain injury questionnaire (QOLIBRI), Rivermead PPCS questionnaire (RPSQ-3, RPSQ-13), and British Columbia post-concussion symptom inventory (BCPSI). fNIRS recordings and clinical questionnaires were completed at baseline, immediately following rTMS (day 14), and at one month (day 45) post-rTMS. An 8-week headache diary documenting frequency and severity was also completed (2 weeks at baseline, during treatment, post-treatment, and 1-month post treatment).

### fNIRS

**Figure 1:**
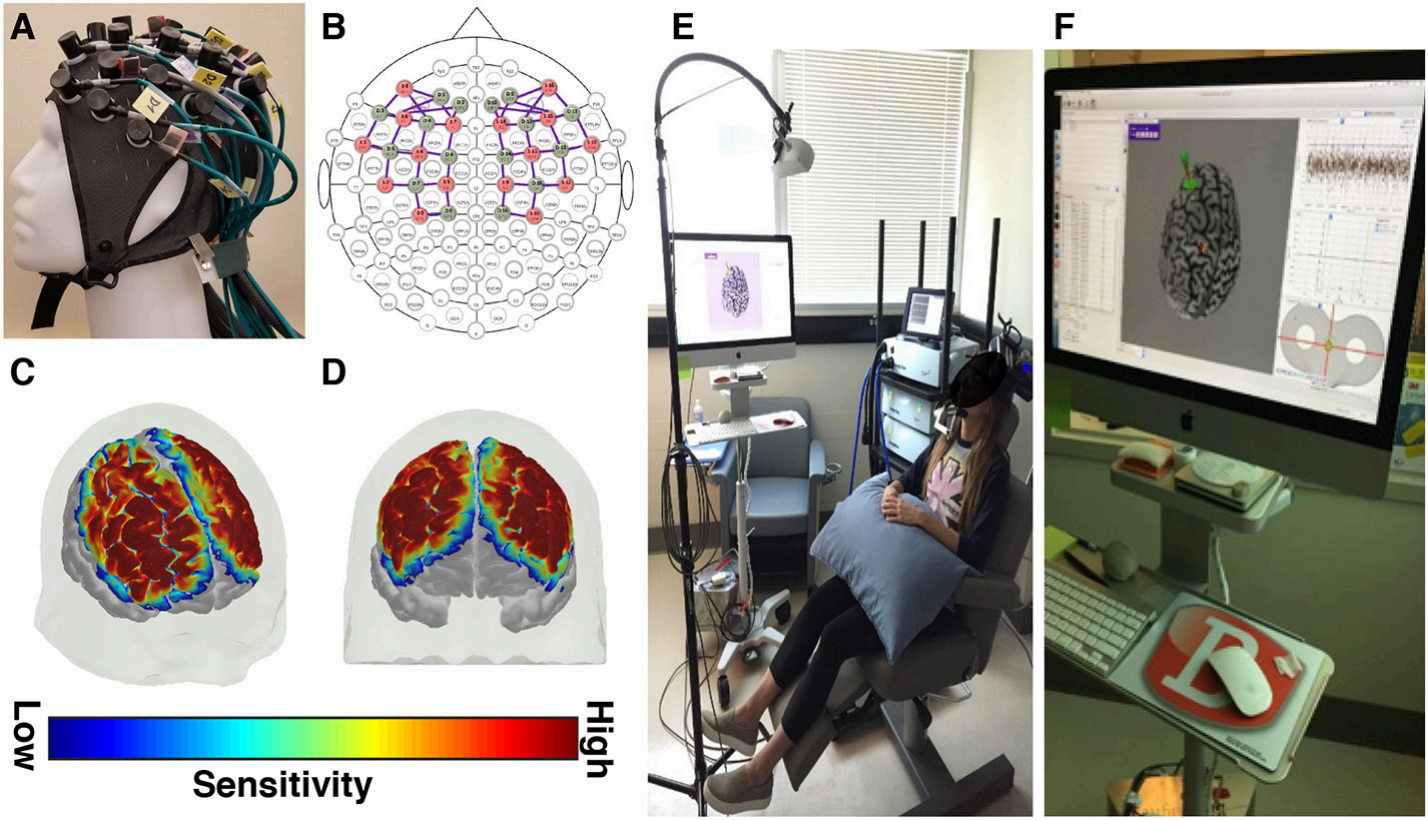
fNIRS and rTMS equipment descriptions. **(A)** A custom fNIRS headcap and **(B)** optode configuration was used. The fNIRS headcap was designed to measure tissue oxygenation over fronto-parietal brain areas, including the dorsolateral prefrontal cortex and the primary motor cortex. **(C,D)** The sensitivity maps (shown here at 2 different angles) depict the areas of the cerebral cortex where tissue oxygenation was recorded, based on the custom optode configuration. These sensitivity maps are created by projecting the simulated photon paths for each fNIRS channel onto a 3D model of the brain. **(E)** rTMS equipment configuration and **(F)** rTMS neuronavigational system.

Functional near infrared spectroscopy (fNIRS) scans were recorded at baseline, immediately following rTMS (day 14), and at one month (day 45) post-rTMS to investigate changes in brain physiology associated with rTMS treatment. fNIRS data were recorded over the frontoparietal cortex at a sampling rate of 3.91 Hz, using the NIRScout fNIRS system (NIRx Medical Technologies, Berlin, Germany; Figure 1). Each recording consisted of a 5 min rest period, followed by a finger tapping exercise, and a graded working memory task, previously described by Hocke et al. (21). The fNIRS data was processed and analyzed for task-evoked activation using an ordinary least squares method of general linear modeling, as implemented in the NIRS Brain AnalyzIR Toolbox (22). See Huppert et al. for a detailed discussion of fNIRS principles, acquisition, and analysis (23).

## Results

### Clinical Scores

Participant 1 reported moderate overall symptom burden at baseline based on symptom scores, with minor improvements in most clinical scores following rTMS (Table 1). Participant 2 had greater symptom burden at baseline, and experienced improvements in all clinical scores, with clinically significant improvements in headache frequency, functional impact, and depression post-rTMS treatment, which persisted at 1-month post-rTMS (day 45; Table 1).

**Table 1:** Clinical questionnaire outcome measures before treatment (day 1), immediately following rTMS (day 14), and at one-month post treatment (day 45).

**Assessments**		**rTMS participant 1**				**rTMS participant 2**			
**Questionnaire**	**MCID**	**Pre-rTMS (Day 1)**	**Post rTMS (Day 14)**	**Follow-up (Day 45)**	**Clinically Important Change**	**Pre-rTMS (Day 1)**	**Post rTMS (Day 14)**	**Follow-up (Day 45)**	**Clinically important change**
Headache Frequency	50% dec./month (15, 24, 25)	28	28	28	–	39	29	16	**+**
Headache Severity	2 (26)	2.75	2.42	2	–	5.56	4.06	4.37	–
MoCA		29	30	26		26	26	28	
HIT-6	8 (27)	64 (severe)	63 (severe)	61 (severe)	–	68 (severe)	65 (severe)	60 (severe)	**+**
PHQ-9	5 (28)	11 (mod.)	10 (mod.)	9 (mild)	–	25 (severe)	4 (minimal)	8 (mild)	**+**
GAD-7		7 (mild)	5 (mild)	6 (mild)		18 (severe)	4 (minimal)	10 (mod.)	
PCL-5		10	11	10		54 (further testing req'd)	16	15	
QOLIBRI		53 (severe)	56 (severe)	66 (mod.)		6 (severe)	53 (severe)	31 (severe)	
RPSQ-3		6	8	7		11	6	6	
RPSQ-13		25	22	22		48	29	22	
BCPSI total		65	42	47		106	38	59	

*Clinical scores improved (headache severity, MoCA, HIT-6, PHQ-9, GAD-7, QOLIBRI, RPSQ, BCPSI) or remained stable (PCL-5, headache frequency) immediately post-rTMS, for both participants. All follow up scores from the one-month assessment (day 45), for headache severity, mood, function and quality of life, improved compared to baseline for both subjects, suggesting persistent effects following rTMS treatment*.

*MCID, minimal clinically important difference; MoCA, Montreal cognitive assessment; HIT-6 (functional impairment), headache impact test-6; PHQ-9 (depression), patient health questionnaire-9; GAD-7 (anxiety), generalized anxiety disorder scale-7; PCL-5 (post-traumatic stress), PTSD Checklist for DSM-5; QOLIBRI, quality of life in brain injury; RPSQ-3, RPSQ-13, Rivermead post-concussion symptom questionnaire; BC-PSI, British Columbia post-concussion symptoms questionnaire*.

Both participants reported decreased headache severity immediately following rTMS and the effect persisted at the 1 month follow up. Headache frequency did not change in participant 1, however, there was a gradual reduction in headache frequency in participant 2. Clinical questionnaire outcomes, including the MoCA, HIT-6, PHQ-9, GAD-7, and QOLIBRI, RPSQ-3, RPSQ-13, and BCPSI all either improved or stayed the same immediately following rTMS treatment for both participants (day 14). Further, follow up scores from the 1-month assessment (day 45) improved compared to baseline for both subjects, suggesting persistent effects on headache severity, function, mood, and quality of life (Table 1). Participant 1 maintained part time work following rTMS and participant 2 was able to return to work at the completion of treatment.

### fNIRS

**Figure 2:**
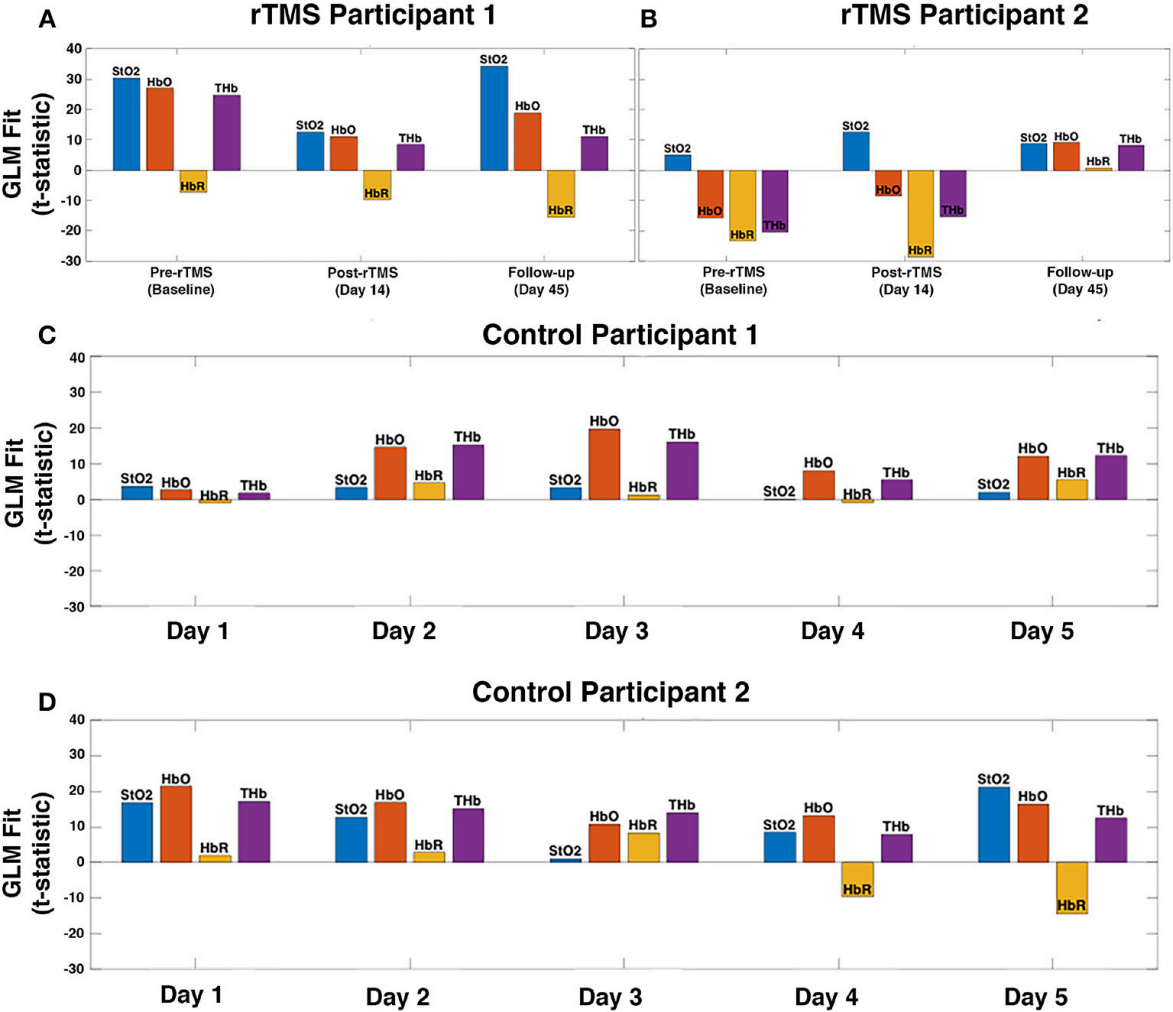
fNIRS measured hemodynamic response to a working memory task in the left DLPFC of patients with persistent post-concussion symptoms (PPCS) who received repetitive transcranial magnetic stimulation (rTMS) treatment **(A,B)**. As an example of the reproducibility of the hemodynamic response pattern to this task, data from two healthy sex-matched controls across five measurement days is presented **(C,D)**. A general linear model (GLM) was used to calculate the location and magnitude of task-evoked changes in oxygen saturation (StO_2_), oxyhemoglobin (HbO), deoxyhemoglobin (HbR), and total hemoglobin (THb) for each participant. **(A)** rTMS participant 1 exhibits normal task-evoked hemodynamic response (characterized by a large increase in HbO) at baseline, as well as post-rTMS (day 14 and follow-up day 45). **(B)** rTMS participant 2 exhibits abnormal task-evoked hemodynamic response (HbO decreased during working memory task execution) at baseline and post-rTMS day 14, however, is normalized 1 month after rTMS treatment (day 45). **(C,D)** As expected, a consistent hemodynamic response of increased oxygenation was observed in the control subjects across all five measurement days.

**Figure 3:**
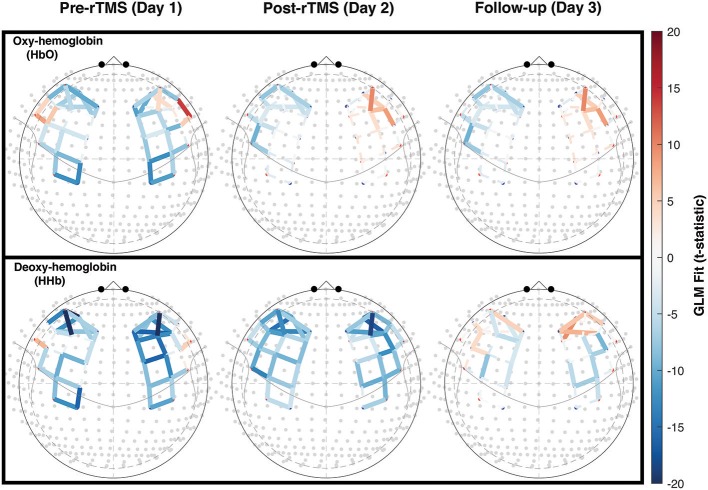
Working memory task-activation color maps for rTMS participant 2, at each time point. Abnormal hemodynamic response (significant deactivation) is detected in the left DLPFC at pre- and post-rTMS timepoints (day 1 and 14) and appears to be normalized (significant activation) by follow-up (day 45). Statistical t-values from the GLM are mapped as red (positive or activation) and blue (negative or deactivation) for each channel pair. Colors increase in intensity as the absolute t-value increases. Solid colored lines represent significance at the level of *p* < 0.05, after correction for false discovery rate. Broken lines represent non-significant changes.

It has been shown previously that working memory tasks evoke a robust hemodynamic response in the DLPFC, characterized by an increase in oxygenated hemoglobin (29, 30). This expected hemodynamic response was observed across 5 measurement days in the controls, highlighting the reproducibility of the activation pattern (Figures 2C,D). In patients with PPCS, participant 1 demonstrated the expected task-evoked hemodynamic response to the working memory task at baseline and both post-rTMS time points (Figure 2A). Interestingly, participant 2 exhibited an *abnormal* fNIRS hemodynamic response to the working memory test (Figures 2B, 3) whereby oxygenated hemoglobin in the left DLPFC was decreased at the baseline time-point. This response appeared to normalize by the 45-day follow-up.

## Discussion

This paper is a preliminary investigation of the feasibility and applicability of using fNIRS to quantitatively assess functional responses to rTMS treatment. Prefrontal cortex rTMS has shown promise as an effective treatment for multiple disorders, particularly depression (10, 11) and headache (12, 31–33) which are both prominent post-concussion symptoms. Consequently, rTMS has been proposed as a treatment for PPCS. Several studies report that rTMS in patients with PPCS may significantly reduce symptoms of headache, depression, dizziness, and improve quality of life (13–15, 34, 35), however these studies are limited by small sample sizes, weak study design, and/or a lack of objective tools to assess treatment response. Furthermore, little is known about the physiological mechanism of how rTMS influences symptoms and function in this population. Therefore, we propose using fNIRS technology to assess possible functional changes in PPCS patients following rTMS intervention.

### Treatment of PPCS With rTMS

In this case study, two participants with PPCS who received 10 sessions of rTMS to the left DLPFC reported positive outcomes following treatment, with participant 2 reporting greater than the minimal clinically important difference (MCID) on several questionnaires. There were many similarities between the two participants with regards to the presence of symptoms that were reported at baseline (i.e., headaches, dizziness, vision changes, neck pain, fatigue, and mood difficulties), although, participant 2 reported overall more severe symptom burden prior to rTMS treatment, which may have contributed to their difference in post-rTMS treatment response.

In particular, at baseline, participant 2 had markedly higher headache frequency (39 vs. 28; participant 2 vs. participant 1, respectively), headache severity (5.56 vs. 2.75), mood symptoms (PHQ-9 depression score: 25 vs. 11; GAD-7 anxiety score: 18 vs. 7; PCL-5 post-traumatic stress score: 54 vs. 10), lower quality of life (QOLIBRI: 6 vs. 53), and more severe post-concussion symptom scores (RPSQ-3: 11 vs. 6; RPSQ-13: 48 vs. 25; BCPSI: 106 vs. 65), in comparison to participant 1 (Table 1). At follow-up (45 days post-rTMS intervention) participant 2 reported clinically significant improvements in headache frequency (59% decrease), functional impact of headaches (HIT-6: 8-point reduction), and depression (PHQ-9: 17-point reduction), as well as a large reduction in global post-concussion symptoms and improved quality of life. On the other hand, participant 1 reported improvement on most clinical questionnaire outcomes following rTMS, however changes were below the MCID (Table 1). This case study, although a limited sample, suggests that rTMS treatment response in PPCS patients is variable, and may relate to baseline function.

Despite persistence of lengthy post-concussion symptoms in both participants, there was an immediate positive effect on self-reported symptoms following rTMS treatment (headache severity, depression, anxiety, cognition, functional impact, quality of life). To date, few studies have investigated rTMS for the treatment of PPCS. Koski et al. performed rTMS to the DLPFC in patients with PPCS>3 months following injury (13). Although there were differences in rTMS stimulation protocol compared to our study (20 sessions, 10 Hz, 110% RMT, 600 pulses), they too found a decline in post-concussion symptom scores post-rTMS, which correlated with increased fMRI task-related activation peaks in the DLPFC.

### fNIRS as a Tool to Explore rTMS Treatment Response

A number of studies report that chronic headache, depression, and mTBI are associated with alterations in prefrontal brain areas, characterized by reduced gray matter volume and/or cortical thickness, changes in frontal white matter microstructure, as well as altered function in studies of task-response and functional connectivity (36–44). Interestingly, many studies report functional and behavioral improvements following prefrontal rTMS treatment in patients with these disorders (14, 15, 34, 35, 45–49). This has led to the hypothesis that rTMS to the prefrontal cortex can alter its function and microstructure, and result in improved outcomes in patients with PPCS. As a cost-effective neuroimaging tool, functional near infrared spectroscopy (fNIRS) is well suited for assessing functional changes following rTMS treatment and has the potential to add to our understanding of the mechanism by which these changes occur.

fNIRS is a functional imaging technology that measures changes in cortical tissue oxygenation which correspond to local changes in brain metabolism, allowing for portable and non-invasive measurement of functional brain changes (16, 17). Our group has previously shown that fNIRS has the potential to detect alterations in brain function associated with concussion injury (21, 50). In addition to our findings, two other studies suggest that fNIRS is sensitive to altered function in the prefrontal cortex of patients with post-concussion symptoms, particularly while performing cognitive tasks such as visual attention or working memory (21, 51, 52). In this case study, we used fNIRS measures of functional activation in the left DLPFC, in response to a working memory task, as a method to assess baseline function and rTMS treatment response. A normal hemodynamic response recorded by fNIRS during a working memory task is characterized by a robust increase in oxygenated hemoglobin and a far less robust decrease (or no change at all) in deoxygenated hemoglobin, in the DLPFC (29, 30). This normal or expected response of a robust increase in oxygenated hemoglobin is evident in the two control subjects across five measurement time-points, highlighting the reproducibility of this measurement (Figures 2C,D). In addition, total hemoglobin (oxygenated + deoxygenated hemoglobin) and oxygen saturation (oxygenated/total hemoglobin) can also be calculated and are both expected to increase with task-activation.

Pre-treatment, participant 2 (high pre-treatment symptom burden) had a decrease in the oxyhemoglobin response to the working memory task, which is an abnormal task-evoked fNIRS hemodynamic response (29, 30). Forty-five days following the start of the rTMS treatment, participant 2's fNIRS response appeared more comparable to the normal response observed in the controls (Figures 2C,D), which paralleled the significant improvement in clinical scores. In participant 1, who had less severe symptom burden at baseline, we observed an expected task-evoked fNIRS hemodynamic response at baseline (similar to control subjects), as well as post-rTMS treatment (Figure 2A).

The exact mechanism by which rTMS treatment improves brain function is still a matter of debate, although one proposed mechanism that has gained traction is the hypothesis that rTMS helps to re-establish connections in areas of the brain that exhibit dysfunctional activity (53, 54). This hypothesis aligns with our preliminary findings in participant two who had a positive treatment response, after exhibiting high symptom burden and altered fNIRS response prior to treatment. Further, the idea that baseline physiological features may, in part, determine rTMS treatment response was recently explored in a cohort of individuals with depression. The authors of this study report that a baseline decrease in the ratio of blood flow in the dorsolateral prefrontal cortex (DLPFC) relative to the ventromedial prefrontal cortex (VMPFC) was predictive of treatment response (55), suggesting that those who experience the greatest treatment-response have decreased DLPFC cerebral blood flow at baseline. Abnormal vascular coupling or network activation may help to explain the unusual reduction in oxyhemoglobin we observed in participant 2.

Considering this is a case study of two participants, we cannot accurately determine the timeline or trajectory of recovery of fNIRS measured brain function, nor can we conclude with confidence that pre-injury function on fNIRS measures relate to treatment response. These preliminary findings do however suggest that fNIRS could be utilized in future studies as a means to better understand the acute and long term effects of rTMS treatment, and support the hypothesis that pre-treatment baseline function may play a role in who responds best to rTMS intervention. Taken together, this two-patient case study highlights a potential role for fNIRS imaging technology to be used as a tool for assessing rTMS treatment response in patients with PPCS.

### Limitations

Potential limitations identified in our study include differences in the mechanism of injury and length of time between concussion to rTMS treatment, for the two participants. Participant 1 received rTMS treatment ~3 years following a sport-related concussion, whereas participant 2 received rTMS treatment 8 months following a motor vehicle accident-related concussion. Pre-injury risk factors for developing post-concussion symptoms, including migraine, depression, life stressors, and personality characteristics may have played a role in persistence of symptoms in the two participants (56). In addition, a limitation of fNIRS is that it is only sensitive to hemodynamic changes in the cerebral cortex, limiting the ability to study deeper brain structures. Further, this study was completed on a small sample size with only one sex (male) represented, and control participants were not age matched to rTMS subjects. Although age-related effects on the magnitude of response to working memory have been observed previously, the direction of the task-effect (increase in oxygenation) does not differ between young and old healthy adults, suggesting the response we observed in participant 2 is unrelated to aging (57). Time since injury and mechanism of injury may factor into rTMS treatment efficacy and should be considered in future trials, along with a larger sample of mTBI patients and including age- and sex-matched controls.

## Conclusion

To our knowledge, this is the first study to utilize functional near infrared spectroscopy (fNIRS) to explore rTMS treatment response in participants with persistent post-concussion symptoms. In this pilot trial, both participants reported symptom amelioration and improved quality of life after rTMS. Interestingly, participant 2, who had more severe symptomatology also showed abnormal oxyhemoglobin (hemodynamic) response to a working memory task as quantified with fNIRS. As participant 2's symptoms improved, the fNIRS hemodynamic response also changed to a more typical or expected pattern (increased oxyhemoglobin) during task response.

This study demonstrates the feasibility of examining rTMS treatment response with fNIRS and suggests the possibility of a measurable relationship between the two technologies. fNIRS may be a sensitive tool to predict response to rTMS in patients with PPCS, providing a first step toward utilizing fNIRS as an objective assessment tool in future rTMS trials. To further evaluate rTMS treatment efficacy and gain a greater understanding of the physiological changes underlying rTMS intervention for PPCS, large longitudinal clinical trials with objective assessments at multiple time points are needed. As a cost-effective portable neuroimaging device, fNIRS is well-suited for this role.

## Ethics Statement

This was a case study of two patients. We received written consent from these two patients in accordance with the University of Calgary Research Ethics board. However, as there was not a protocol, ethics was not approved for the protocol.

## Author Contributions

Conception and study design were completed by CCD, JMS, CTD, and JFD. Data collection was done by CCD, IO, JMS, and EP. Data analysis and interpretation was performed by CCD, JFD, JMS, and CTD. Drafting the article was done by JMS and CCD. Critical revision of the article was completed by all authors. Final approval of the version to be published was done by JMS, CCD, and CTD.

### Conflict of Interest Statement

The authors declare that the research was conducted in the absence of any commercial or financial relationships that could be construed as a potential conflict of interest.

## References

[1] Taylor CA Bell JM Breiding MJ Xu L. Traumatic brain injury-related emergency department visits, hospitalizations, and deaths - United States, 2007 and 2013. Morbidity Mortality Wkly Rep Surv Summ. (2017) 66:1–16. 10.15585/mmwr.ss6609a1 28301451 PMC5829835

[2] Gardner RC Yaffe K. Epidemiology of mild traumatic brain injury and neurodegenerative disease. Mol Cell Neurosci. (2015) 66:75–80. 10.1016/j.mcn.2015.03.001 25748121 PMC4461453

[3] Cassidy JD Carroll L Peloso P Borg J Von Holst H Holm L Incidence, risk factors and prevention of mild traumatic brain injury: results of the WHO Collaborating Centre Task Force on Mild Traumatic Brain Injury. J Rehab Med. (2004) 43:28–60. 10.1080/16501960410023732 15083870

[4] McMahon P Hricik A Yue JK Puccio AM Inoue T Lingsma HF. Symptomatology and functional outcome in mild traumatic brain injury: results from the prospective track-TBI study. J Neurotrauma. (2014) 31:26–33. 10.1089/neu.2013.2984 23952719 PMC3880097

[5] Kashluba S Casey JE Paniak C. Evaluating the utility of ICD-10 diagnostic criteria for postconcussion syndrome following mild traumatic brain injury. J Int. Neuropsychol Soc. (2006) 12:111–8. 10.1017/S1355617706060036 16433950

[6] World Health Organization The ICD-10 Classification of Mental and Behavioural Disorders: Clinical Descriptions and Diagnostic Guidelines, Section F07.2. Geneva: World Health Organization (1992).

[7] Boake C McCauley SR Levin HS Pedroza C Contant CF Song JX. Diagnostic criteria for postconcussional syndrome after mild to moderate traumatic brain injury. J Neuropsychiatry Clin Neurosci. (2005) 17:350–6. 10.1176/jnp.17.3.350 16179657

[8] Mayer AR Quinn DK Master CL. The spectrum of mild traumatic brain injury: a review. Neurology. (2017) 89:623–32. 10.1212/WNL.0000000000004214 28701496 PMC5562956

[9] Hallett M Transcranial magnetic stimulation and the human brain. Nature. (2000) 406:147–50. 10.1038/35018000 10910346

[10] O'Reardon JP Solvason HB Janicak PG Sampson S Isenberg KE Nahas Z. Efficacy and safety of transcranial magnetic stimulation in the acute treatment of major depression: a multisite randomized controlled trial. Biol Psychiatry. (2007) 62:1208–16. 10.1016/j.biopsych.2007.01.018 17573044

[11] Brunoni AR Chaimani A Moffa AH Razza LB Gattaz WF Daskalakis ZJ. Repetitive transcranial magnetic stimulation for the acute treatment of major depressive episodes: a systematic review with network meta-analysis. JAMA Psychiatry. (2017) 74:143–52. 10.1001/jamapsychiatry.2016.3644 28030740

[12] Lipton RB Dodick DW Silberstein SD Saper JR Aurora SK Pearlman SH. Single-pulse transcranial magnetic stimulation for acute treatment of migraine with aura: a randomised, double-blind, parallel-group, sham-controlled trial. Lancet Neurol. (2010) 9:373–80. 10.1016/S1474-4422(10)70054-5 20206581

[13] Koski L Kolivakis T Yu C Chen JK Delaney S Ptito A. Noninvasive brain stimulation for persistent postconcussion symptoms in mild traumatic brain injury. J Neurotrauma. (2015) 32:38–44. 10.1089/neu.2014.3449 24955920

[14] Leung A Metzger-Smith V He Y Cordero J Ehlert B Song D. Left dorsolateral prefrontal cortex rTMS in alleviating MTBI related headaches and depressive symptoms. Neuromodulation. (2017) 21:390–401. 10.1111/ner.12615 28557049

[15] Leung A Shukla S Fallah A Song D Lin L Golshan S. Repetitive transcranial magnetic stimulation in managing mild traumatic brain injury-related headaches. Neuromodulation. (2016) 19:133–41. 10.1111/ner.12364 26555886

[16] Scholkmann F Kleiser S Metz AJ Zimmermann R Mata Pavia J Wolf U. A review on continuous wave functional near-infrared spectroscopy and imaging instrumentation and methodology. Neuroimage. (2014) 85(Pt 1):6–27. 10.1016/j.neuroimage.2013.05.004 23684868

[17] Kleinschmidt A Obrig H Requardt M Merboldt KD Dirnagl U Villringer A. Simultaneous recording of cerebral blood oxygenation changes during human brain activation by magnetic resonance imaging and near-infrared spectroscopy. J Cereb Blood Flow Metab. (1996) 16:817–26. 10.1097/00004647-199609000-00006 8784226

[18] McCrory P Meeuwisse W Dvorak J Aubry M Bailes J Broglio S Consensus statement on concussion in sport-the 5(th) international conference on concussion in sport held in Berlin, October 2016. Br J Sports Med. (2017) 51:838–47. 10.1136/bjsports-2017-097699 28446457

[19] Dodick DW Turkel CC DeGryse RE Aurora SK Silberstein SD Lipton RB. OnabotulinumtoxinA for treatment of chronic migraine: pooled results from the double-blind, randomized, placebo-controlled phases of the PREEMPT clinical program. Headache. (2010) 50:921–36. 10.1111/j.1526-4610.2010.01678.x 20487038

[20] Ruff RM Iverson GL Barth JT Bush SS Broshek DK Methodological issues and research recommendations for mild traumatic brain injury: the WHO Collaborating Centre Task Force on Mild Traumatic Brain Injury. J Rehabil Med. (2004) 43:113–25. 10.1080/16501960410023877 15083875

[21] Hocke LM Duszynski CC Debert CT Dleikan D Dunn JF. Reduced functional connectivity in adults with persistent post-concussion symptoms: a functional near-infrared spectroscopy study. J Neurotrauma. (2018) 35:1224–32. 10.1089/neu.2017.5365 29373947 PMC5962910

[22] Santosa H Zhai X Fishburn F Huppert T The NIRS brain AnalyzIR toolbox. Algorithms. (2018) 11:73 10.3390/a11050073 PMC11218834 38957522

[23] Huppert TJ Diamond SG Franceschini MA Boas DA. HomER: a review of time-series analysis methods for near-infrared spectroscopy of the brain. Appl Optics. (2009) 48:D280–8. 10.1364/AO.48.00D280 19340120 PMC2761652

[24] Kalita J Laskar S Bhoi SK Misra UK. Efficacy of single versus three sessions of high rate repetitive transcranial magnetic stimulation in chronic migraine and tension-type headache. J Neurol. (2016) 263:2238–46. 10.1007/s00415-016-8257-2 27541044

[25] Schoenen J. Guidelines for trials of drug treatments in tension-type headache. First edition: International Headache Society Committee on Clinical Trials. Cephalalgia. (1995) 15:165–79. 10.1046/j.1468-2982.1995.015003165.x 7553803

[26] Farrar JT Young JP Jr LaMoreaux L Werth JL Poole RM. Clinical importance of changes in chronic pain intensity measured on an 11-point numerical pain rating scale. Pain. (2001) 94:149–58. 10.1016/S0304-3959(01)00349-9 11690728

[27] Castien RF Blankenstein AH Windt DA Dekker J. Minimal clinically important change on the Headache Impact Test-6 questionnaire in patients with chronic tension-type headache. Cephalalgia. (2012) 32:710–4. 10.1177/0333102412449933 22707519

[28] Lowe B Schenkel I Carney-Doebbeling C Gobel C. Responsiveness of the PHQ-9 to psychopharmacological depression treatment. Psychosomatics. (2006) 47:62–7. 10.1176/appi.psy.47.1.62 16384809

[29] Leon-Dominguez U Martin-Rodriguez JF Leon-Carrion J. Executive n-back tasks for the neuropsychological assessment of working memory. Behav Brain Res. (2015) 292:167–73. 10.1016/j.bbr.2015.06.002 26068585

[30] Owen AM McMillan KM Laird AR Bullmore E. N-back working memory paradigm: a meta-analysis of normative functional neuroimaging studies. Hum Brain Mapp. (2005) 25:46–59. 10.1002/hbm.20131 15846822 PMC6871745

[31] Brighina F Piazza A Vitello G Aloisio A Palermo A Daniele O. rTMS of the prefrontal cortex in the treatment of chronic migraine: a pilot study. J Neurol Sci. (2004) 227:67–71. 10.1016/j.jns.2004.08.008 15546593

[32] Misra UK Kalita J Bhoi SK. High-rate repetitive transcranial magnetic stimulation in migraine prophylaxis: a randomized, placebo-controlled study. J Neurol. (2013) 260:2793–801. 10.1007/s00415-013-7072-2 23963471

[33] Stilling JM Monchi O Amoozegar F Debert CT. Transcranial magnetic and direct current stimulation (TMS/tDCS) for the treatment of headache: a systematic review. Headache. (2019) 59:339–57. 10.1111/head.13479 30671941

[34] Leung A Fallah A Shukla S Lin L Tsia A Song D rTMS in alleviating mild TBI related headaches–a case series. Pain Phys. (2016) 19:E347–54. 26815263

[35] Paxman E Stilling J Mercier L Debert CT. Repetitive transcranial magnetic stimulation (rTMS) as a treatment for chronic dizziness following mild traumatic brain injury. BMJ Case Rep. (2018) 2018:bcr-2018-226698. 10.1136/bcr-2018-226698 30396889 PMC6229180

[36] Eierud C Craddock RC Fletcher S Aulakh M King-Casas B Kuehl D. Neuroimaging after mild traumatic brain injury: review and meta-analysis. Neuroimage Clin. (2014) 4:283–94. 10.1016/j.nicl.2013.12.009 25061565 PMC4107372

[37] Rogers MA Kasai K Koji M Fukuda R Iwanami A Nakagome K. Executive and prefrontal dysfunction in unipolar depression: a review of neuropsychological and imaging evidence. Neurosci Res. (2004) 50:1–11. 10.1016/j.neures.2004.05.003 15288493

[38] Yang Q Wang Z Yang L Xu Y Chen LM. Cortical thickness and functional connectivity abnormality in chronic headache and low back pain patients. Hum Brain Mapp. (2017) 38:1815–32. 10.1002/hbm.23484 28052444 PMC6867133

[39] Chen WT Chou KH Lee PL Hsiao FJ Niddam DM Lai KL. Comparison of gray matter volume between migraine and “strict-criteria” tension-type headache. J Headache Pain. (2018) 19:4. 10.1186/s10194-018-0834-6 29335889 PMC5768588

[40] Ma M Zhang J Chen N Guo J Zhang Y He L. Exploration of intrinsic brain activity in migraine with and without comorbid depression. J Headache Pain. (2018) 19:48. 10.1186/s10194-018-0876-9 29943098 PMC6020083

[41] George MS Ketter TA Post RM Prefrontal cortex dysfunction in *clinical depression*. Depression. (1994) 2:59–72. 10.1002/depr.3050020202

[42] Leung A Yang E Lim M Metzger-Smith V Theilmann R Song D. Pain-related white matter tract abnormalities in mild traumatic brain injury patients with persistent headache. Mol Pain. (2018) 14:1744806918810297. 10.1177/1744806918810297 30324850 PMC6311536

[43] Sacchet MD Gotlib IH. Myelination of the brain in major depressive disorder: an *in vivo* quantitative magnetic resonance imaging study. Sci Rep. (2017) 7:2200. 10.1038/s41598-017-02062-y 28526817 PMC5438403

[44] Obermann M Nebel K Schumann C Holle D Gizewski ER Maschke M. Gray matter changes related to chronic posttraumatic headache. Neurology. (2009) 73:978–83. 10.1212/WNL.0b013e3181b8791a 19770474

[45] Blumberger DM Vila-Rodriguez F Thorpe KE Feffer K Noda Y Giacobbe P. Effectiveness of theta burst versus high-frequency repetitive transcranial magnetic stimulation in patients with depression (THREE-D): a randomised non-inferiority trial. Lancet. (2018) 391:1683–92. 10.1016/S0140-6736(18)30295-2 29726344

[46] Conforto AB Amaro E Gonçalves AL Mercante JP Guendler VZ Ferreira JR. Randomized, proof-of-principle clinical trial of active transcranial magnetic stimulation in chronic migraine. Cephalalgia. (2014) 34:464–72. 10.1177/0333102413515340 24326236

[47] Siddiqi SH Trapp NT Hacker CD Laumann TO Kandala S Hong X. Repetitive transcranial magnetic stimulation with resting state network targeting for treatment-resistant depression in traumatic brain injury: a randomized, controlled, double blinded pilot study. J Neurotrauma. (2018) 36:1361–74. 10.1089/neu.2018.5889 30381997 PMC6909726

[48] Reti IM Schwarz N Bower A Tibbs M Rao V. Transcranial magnetic stimulation: a potential new treatment for depression associated with traumatic brain injury. Brain Inj. (2015) 29:789–97. 10.3109/02699052.2015.1009168 25950260

[49] Hsu JH Daskalakis ZJ Blumberger DM. An update on repetitive transcranial magnetic stimulation for the treatment of co-morbid pain and depressive symptoms. Curr Pain Headache Rep. (2018) 22:51. 10.1007/s11916-018-0703-7 29904802

[50] Urban KJ Barlow KM Jimenez JJ Goodyear BG Dunn JF. Functional near-infrared spectroscopy reveals reduced interhemispheric cortical communication after pediatric concussion. J Neurotrauma. (2015) 32:833–40. 10.1089/neu.2014.3577 25387354 PMC4449632

[51] Kontos AP Huppert TJ Beluk NH Elbin RJ Henry LC French J. Brain activation during neurocognitive testing using functional near-infrared spectroscopy in patients following concussion compared to healthy controls. Brain Imag Behav. (2014) 8:621–34. 10.1007/s11682-014-9289-9 24477579 PMC5080617

[52] Wu Z Mazzola CA Catania L Owoeye O Yaramothu C Alvarez T. Altered cortical activation and connectivity patterns for visual attention processing in young adults post-traumatic brain injury: a functional near infrared spectroscopy study. CNS Neurosci Therapeut. (2018) 24:539–48. 10.1111/cns.12811 29359534 PMC6490005

[53] Anderson RJ Hoy KE Daskalakis ZJ Fitzgerald PB. Repetitive transcranial magnetic stimulation for treatment resistant depression: Re-establishing connections. Clin Neurophysiol. (2016) 127:3394–405. 10.1016/j.clinph.2016.08.015 27672727

[54] Taïb S Arbus C Sauvaget A Sporer M Schmitt L Yrondi A. How does repetitive transcranial magnetic stimulation influence the brain in depressive disorders?: A review of neuroimaging magnetic resonance imaging studies. J ECT. (2018) 34:79–86. 10.1097/YCT.0000000000000477 29324522

[55] Kito S Hasegawa T Koga Y. Cerebral blood flow ratio of the dorsolateral prefrontal cortex to the ventromedial prefrontal cortex as a potential predictor of treatment response to transcranial magnetic stimulation in depression. Brain Stimul. (2012) 5:547–53. 10.1016/j.brs.2011.09.004 22019081

[56] Wäljas M Iverson GL Lange RT Hakulinen U Dastidar P Huhtala H. A prospective biopsychosocial study of the persistent post-concussion symptoms following mild traumatic brain injury. J Neurotrauma. (2015) 32:534–47. 10.1089/neu.2014.3339 25363626

[57] Rajah MN D'Esposito M. Region-specific changes in prefrontal function with age: a review of PET and fMRI studies on working and episodic memory. Brain. (2005) 128:1964–83. 10.1093/brain/awh608 16049041

